# Muscle fiber hypertrophy in response to 6 weeks of high-volume resistance training in trained young men is largely attributed to sarcoplasmic hypertrophy

**DOI:** 10.1371/journal.pone.0215267

**Published:** 2019-06-05

**Authors:** Cody T. Haun, Christopher G. Vann, Shelby C. Osburn, Petey W. Mumford, Paul A. Roberson, Matthew A. Romero, Carlton D. Fox, Christopher A. Johnson, Hailey A. Parry, Andreas N. Kavazis, Jordan R. Moon, Veera L. D. Badisa, Benjamin M. Mwashote, Victor Ibeanusi, Kaelin C. Young, Michael D. Roberts

**Affiliations:** 1 Department of Exercise Science, LaGrange College, LaGrange, GA, United States of America; 2 School of Kinesiology, Auburn University, Auburn, AL, United States of America; 3 School of Medicine, University of Alabama Birmingham, Birmingham, AL, United States of America; 4 Impedimed, Carlsbad, CA, United States of America; 5 School of the Environment, Florida A&M University, Tallahassee, FL, United States of America; 6 Department of Cell Biology and Physiology, Edward Via College of Osteopathic Medicine—Auburn Campus, Auburn, AL, United States of America; University of Minnesota Medical School, UNITED STATES

## Abstract

Cellular adaptations that occur during skeletal muscle hypertrophy in response to high-volume resistance training are not well-characterized. Therefore, we sought to explore how actin, myosin, sarcoplasmic protein, mitochondrial, and glycogen concentrations were altered in individuals that exhibited mean skeletal muscle fiber cross-sectional area (fCSA) hypertrophy following 6 weeks of high-volume resistance training. Thirty previously resistance-trained, college-aged males (mean ± standard deviation: 21±2 years, 5±3 training years) had vastus lateralis (VL) muscle biopsies obtained prior to training (PRE), at week 3 (W3), and at week 6 (W6). Muscle tissue from 15 subjects exhibiting PRE to W6 VL mean fCSA increases ranging from 320–1600 μm^2^ was further interrogated using various biochemical and histological assays as well as proteomic analysis. Seven of these individuals donated a VL biopsy after refraining from training 8 days following the last training session (W7) to determine how deloading affected biomarkers. The 15 fCSA hypertrophic responders experienced a +23% increase in mean fCSA from PRE to W6 (p<0.001) and, while muscle glycogen concentrations remained unaltered, citrate synthase activity levels decreased by 24% (p<0.001) suggesting mitochondrial volume decreased. Interestingly, repeated measures ANOVAs indicated that p-values approached statistical significance for both myosin and actin (p = 0.052 and p = 0.055, respectively), and forced post hoc tests indicated concentrations for both proteins decreased ~30% from PRE to W6 (p<0.05 for each target). Phalloidin-actin staining similarly revealed actin concentrations per fiber decreased from PRE to W6. Proteomic analysis of the sarcoplasmic fraction from PRE to W6 indicated 40 proteins were up-regulated (p<0.05), KEGG analysis indicated that the glycolysis/gluconeogenesis pathway was upregulated (FDR sig. <0.001), and DAVID indicated that the following functionally-annotated pathways were upregulated (FDR value <0.05): a) glycolysis (8 proteins), b) acetylation (23 proteins), c) gluconeogenesis (5 proteins) and d) cytoplasm (20 proteins). At W7, sarcoplasmic protein concentrations remained higher than PRE (+66%, p<0.05), and both actin and myosin concentrations remained lower than PRE (~-50%, p<0.05). These data suggest that short-term high-volume resistance training may: a) reduce muscle fiber actin and myosin protein concentrations in spite of increasing fCSA, and b) promote sarcoplasmic expansion coincident with a coordinated up-regulation of sarcoplasmic proteins involved in glycolysis and other metabolic processes related to ATP generation. Interestingly, these effects seem to persist up to 8 days following training.

## Introduction

Weeks to months of resistance training increases skeletal muscle mean fiber cross-sectional area (fCSA) [1–10]. Tracer studies have also demonstrated that a single bout of resistance exercise increases muscle protein synthesis (MPS) and myofibrillar protein synthesis (MyoPS) rates up to 72 hours post-exercise (reviewed in [11–13]). The ingestible deuterium oxide tracer has enabled scientists to measure integrated MPS and MyoPS for prolonged periods [14, 15], and results have suggested that rates are also elevated weeks into training. Such findings have led to a general consensus that resistance training-induced skeletal muscle hypertrophy occurs via: a) increased myofibrillar protein accretion at the cellular level, and b) an increase in muscle fiber size and diameter due to said protein accretion.

While the aforementioned model is logical, there are human studies which challenge this model. For instance, a study which examined muscle fiber ultrastructural alterations in humans in response to isotonic, isometric, and run training reported decreases in the number of myofibrils per muscle fiber area across all training conditions as assessed by transmission electron microscopy (TEM) [16]. Another study utilizing TEM [17] similarly reported space occupied by myofibrils decreased and space occupied by sarcoplasmic area increased in previously untrained humans following six months of resistance training in lieu of significant increases in fCSA. A similar study utilizing TEM [18] reported that 18 weeks of resistance training resulted in a ~15% decrease in space occupied by myofibrils in both heart failure patients and healthy controls. Our laboratory recently reported that untrained college-aged males with high pre-training myofibrillar protein concentrations, as assessed through biochemical methods, experienced the largest decrease in myofibrillar protein concentrations following 12 weeks of resistance training [19]. Hence, these findings collectively suggest that the hypertrophic response to resistance training may include fiber growth through sarcoplasmic expansion (e.g., intracellular fluid, sarcoplasmic proteins, and glycogen) prior to the accretion of contractile proteins. Upon a comprehensive review of the literature, we recently defined sarcoplasmic hypertrophy as “…*an increase in the volume of the sarcolemma and/or sarcoplasm accompanied by an increase in the volume of mitochondria*, *sarcoplasmic reticulum*, *t-tubules*, *and/or sarcoplasmic enzyme or substrate content*” [20]. However, we also noted a severe lack of research into this area and persuaded further exploration.

We recently published a study in which 31 well-trained college-aged males completed 6 weeks of high-volume resistance training under different nutritional paradigms [21]. In light of the above, the purpose of the current study was to delineate how this training program affected actin and myosin protein concentrations, sarcoplasmic protein concentrations, glycogen concentrations, and mitochondrial volume in 15 individuals which exhibited notable increases in mean fCSA. Per the supporting literature above, we hypothesized that individuals experiencing notable fCSA increases would experience a decrease in myosin and actin concentrations, a decrease in citrate synthase activity (indicative of decreased mitochondrial content), and either no change or an increase in sarcoplasmic protein and glycogen concentrations.

## Materials and methods

### Ethical approval and study design

Prior to engaging in data collection, this study was approved by the Institutional Review Board at Auburn University (Protocol #17–425 MR 1710; IRB contact: irbadmin@auburn.edu) and conformed to the standards set by the latest revision of the Declaration of Helsinki. Resistance-trained college-aged males from the local community were recruited to participate in this study. Participants provided both verbal and written informed consent, and completed a medical history form prior to screening. Two primary criteria were used to establish training status: a) greater than one year of resistance training (self-reported), and b) a back squat one repetition maximum (1RM) ≥1.5× body mass.

### Establishment of mean fCSA responders

We recently analyzed serial muscle sections from 26 participants and reported that the standard error of measurement for detecting fCSA changes in our laboratory is ±459 μm^2^ [20]. Out of the 31 participants in the original dataset, only 30 participants yielded tissue suitable for immunohistochemistry, and only 11 of these participants experienced >459 μm^2^ increase in mean fCSA. Thus, in order to increase statistical power and considering the exploratory nature of this work, we analyzed specimens from a total of 15 subjects demonstrating notably positive PRE to W6 mean fCSA increases ranging from 320–1600 μm^2^. In the original study, participants were partitioned to one of three nutritional supplement groups including: a) daily single dose of whey protein (WP, 25g/d; n = 10), b) daily single dose of maltodextrin (MALTO, 30 g/d; n = 10), or c) graded dose of WP (GWP, 25–150 g/d from weeks 1–6; n = 11). In the 15 participants experiencing mean fCSA increases >320 μm^2^ there was practically equal representation of participants from each of the supplement groups (MALTO n = 6, WP n = 4, GWP n = 5) suggesting that results were likely not influenced from nutritional supplementation. The self-reported resistance training age of the 15 fCSA responders was 5.5±2.8 years, three repetition maximum (3RM) barbell back squat strength prior to the study was 134±22 kg, and 3RM-to-body mass ratio was 1.57±0.24. Readers are referred to Haun et al. [21] for more in-depth descriptions of the supplementation details and nutritional recommendations employed.

### PRE, W3, and W6 testing sessions

Prior to training (PRE), following week 3 (W3), and following week 6 (W6) participants arrived for testing batteries in an overnight fasted condition. At W3 and W6, testing sessions occurred 24 hours following Friday workouts. Testing procedures pertinent to this dataset are described below, although other assessments were performed and described in greater detail elsewhere [21].

#### Urine specific gravity testing for adequate hydration

At the beginning of each testing session, participants submitted a urine sample (~5 mL) to assess urine specific gravity (USG) levels (1.005–1.020 ppm) using a handheld refractometer (ATAGO; Bellevue, WA, USA) as a measure of hydration status. Participants with a USG ≥1.020 were asked to consume 400 mL tap water and were re-tested 20 minutes thereafter.

#### Muscle tissue and blood collection

Muscle biopsies were collected using a 5-gauge needle under local anesthesia as previously described [21]. Immediately following tissue procurement, ~20–40 mg of tissue was embedded in cryomolds containing optimal cutting temperature (OCT) media (Tissue-Tek, Sakura Finetek Inc; Torrence, CA, USA). Embedding was performed according to methods previously published by our laboratory [22] whereby tissue was placed in cryomolds for cross sectional slicing in a non-stretched state prior to rapid freezing. Cryomolds were then frozen using liquid nitrogen-cooled isopentane and subsequently stored at -80°C until histological staining for determination of mean fCSA and actin protein per fiber as described below. The remaining tissue was teased of blood and connective tissue, wrapped in pre-labelled foils, flash frozen in liquid nitrogen, and subsequently stored at -80°C until molecular analyses were performed as described below. Venous blood samples were also collected into a 5 mL serum separator tube (BD Vacutainer, Franklin Lakes, NJ, USA) during the waiting period for anesthesia to take effect for creatine kinase (CK) activity assessment described in greater detail below.

### Resistance training protocol

Participants were familiarized with the design of training and technical parameters during testing of 3RMs which occurred 3–7 days prior to PRE testing and training initiation. Strict technical parameters were employed for testing to ensure accurate reflections of strength under direct supervision of research staff holding the Certified Strength and Conditioning Specialist Certification from the National Strength and Conditioning Association.

Following the PRE testing battery and 3RM testing, RT occurred 3 days per week. Loads corresponding to 60% 1RM, based on 3RM testing, were programmed for each set of each exercise. Sets of 10 repetitions were programmed for each set of each exercise throughout the study. Exercises were completed one set at a time, in the following order during each training session: days 1 and 3 each week–barbell (BB) back squat, BB bench press, BB stiff-legged deadlift, and an underhand grip cable machine pulldown exercise designed to target the elbow flexors and latissimus dorsi muscles (lat pulldown); day 2 of each week–BB back squat, BB overhead press, BB stiff-legged deadlift, and lat pulldown. The 3 day per week protocol involved a progressive increase from 10 sets per week to 32 sets per week for each exercise. Thus, on the last week of training participants performed 32 sets of 10 repetitions of BB back squats, 32 sets of 10 repetitions of BB bench press and overhead press combined, 32 sets of 10 repetitions of BB stiff-legged deadlift, and 32 sets of 10 repetitions of lat pulldowns. Readers are referred to Haun et al. [21] for more in-depth descriptions of training.

### Molecular analyses

Pulverized tissue was used for Western blotting, citrate synthase activity, 20S proteasome activity, and glycogen concentration assays. Tissue foils were removed from -80°C and tissue was crushed on a liquid nitrogen-cooled ceramic mortar with a ceramic pestle. Powdered tissue was weighed using an analytical scale sensitive to 0.0001 g (Mettler-Toledo; Columbus, OH, USA), and placed in 1.7 mL polypropylene tubes containing 500 μL of ice-cold general cell lysis buffer [20 mM Tris-HCl (pH 7.5), 150 mM NaCl, 1 mM Na_2_EDTA, 1 mM EGTA, 1% Triton; Cell Signaling, Danvers, MA, USA] pre-stocked with protease and Tyr/Ser/Thr phosphatase inhibitors (2.5 mM sodium pyrophosphate, 1 mM β-glycerophosphate, 1 mM Na_3_VO_4_, 1 μg/mL leupeptin). Samples were then homogenized by hand using tight-fitting micropestles, insoluble proteins were removed via centrifugation at 500 *g* for 5 minutes, and supernatants (whole-tissue sample lysates) were stored at -80°C and used for further molecular analyses described below.

#### Protein concentration determination

Whole-tissue sample lysates were batch process-assayed for total protein content using a BCA Protein Assay Kit (Thermo Fisher Scientific; Waltham, MA, USA). These lysates were used for Western blotting and enzyme assays described below.

#### Western blotting

Lysates were then prepared for Western blotting using 4x Laemmli buffer at 1 μg/μL and boiled for 5 minutes at 100°C. Samples (15 μL) were loaded onto pre-casted gradient (4–15%) SDS-polyacrylamide gels (Bio-Rad Laboratories; Hercules, CA, USA) and subjected to electrophoresis at 180 V for 60 minutes using pre-made 1x SDS-PAGE running buffer (Ameresco; Framingham, MA, USA). Proteins were then transferred (200 mA for 2 hours) to polyvinylidene difluoride membranes (Bio-Rad Laboratories), Ponceau stained, and imaged to ensure equal protein loading between lanes. Membranes were then blocked for 60 minutes at room temperature with 5% nonfat milk powder in Tris-buffered saline with 0.1% Tween-20 (TBST; Ameresco). Rabbit anti-human ubiquitin (1:1,000 dilution; catalog #: 3933; Cell Signaling) in TBST with 5% bovine serum albumin (BSA) was added to membranes and incubated overnight at 4°C. The following day, membranes were washed and incubated with horseradish peroxidase-conjugated anti-rabbit IgG (catalog #: 7074; Cell Signaling) in TBST (1:2,000 dilution) with 5% BSA at room temperature for 1 hour. Membranes were developed using an enhanced chemiluminescent reagent (Luminata Forte HRP substrate; EMD Millipore, Billerica, MA, USA), and band densitometry was performed using a gel documentation system with associated software (UVP, Upland, CA, USA). Whole-lane densitometry values for poly-ubiquinated proteins of each subject at each time point were divided by whole-lane Ponceau densities in order to derive relative expression levels.

#### Citrate synthase activity assay

Whole-tissue sample lysates were batch processed for citrate synthase activity as previously described [19], and this marker was used as a surrogate for mitochondrial content according to previous literature suggesting citrate synthase activity highly correlates with electron micrograph images of mitochondrial content in skeletal muscle (r = 0.84, p<0.001) [23]. The assay utilized is based on the reduction of 5, 50-dithiobis (2-nitrobenzoic acid) (DTNB) at 412 nm (extinction coefficient 13.6 mmol/L/cm) coupled to the reduction of acetyl-CoA by the citrate synthase reaction in the presence of oxaloacetate. Briefly, 12.5 μg of whole-tissue sample lysates were added to a mixture composed of 0.125 mol/L Tris-HCl (pH 8.0), 0.03 mmol/L acetyl-CoA, and 0.1 mmol/L DTNB. The reaction was initiated by the addition of 5 μL of 50 mM oxaloacetate and the absorbance change was recorded for one minute. The average coefficient of variation values for all duplicates was 5.2%.

#### 20S proteasome activity assay

Protein from whole-tissue sample lysates (40 μg) were batch processed for 20S proteasome activity using commercially available fluorometric kits (catalog #: APT280; Millipore Sigma; Burlington, MA, USA) per the manufacturer’s instructions. Assay readings are presented as raw fluorometric values as previously described by our laboratory [24]. The average coefficient of variation values for all duplicates was 8.7%.

#### Glycogen assay

Whole-tissue sample lysates were batch processed for glycogen determination using commercially-available fluorometric kits (catalog #: MAK016; Millipore Sigma) as per the manufacturer’s instructions. This assay was piloted with whole-tissue sample lysates, frozen wet tissue, and lyophilized tissue, and all three methods yielded similar quantitative results. Thus, given that whole-tissue sample lysates were available for most participants, we opted to assay this tissue fraction. A standard curve was used to determine glycogen content of whole-tissue sample lysates, this value was multiplied by the starting cell lysis buffer volume (500 μL) to derive total glycogen content per sample, and the resultant value was divided by input wet muscle weights to obtain nmol glycogen/mg wet muscle weight. The average coefficient of variation values for all duplicates was 9.8%.

### Assessment of sarcoplasmic protein, myosin, and actin

Myofibrillar and sarcoplasmic protein isolations were performed based on the methods of Goldberg’s laboratory [25]. Briefly, frozen wet powdered muscle (~8–20 mg) was weighed, placed into 1.7 mL polypropylene tubes with ventilation holes drilled on the caps. Samples were freeze-dried using a laboratory-grade freeze-drying device (FreeZone 2.5, Labconco; Kansas City, MO, USA). Freeze dry settings were -50°C for the condenser and a vacuum setting of 0.1 mBar, and samples were placed in the condenser well for 4 hours under vacuum pressure. Following freeze drying, muscle was re-weighed on an analytical scale to determine muscle fluid content, and placed in 1.7 mL polypropylene tubes containing 190 μL of ice cold homogenizing buffer (20 mM Tris-HCl, pH 7.2, 5 mM EGTA, 100 mM KCl, 1% Triton-X 100). Samples were homogenized on ice using tight-fitting pestles, and centrifuged at 3000 *g* for 30 minutes at 4°C. Supernatants (sarcoplasmic fraction) were collected, placed in 1.7 mL polypropylene tubes, and stored at -80°C. The resultant myofibril pellet was resuspended in homogenizing buffer, and samples were centrifuged at 3000 *g* for 10 minutes at 4°C. Resultant supernatants from this step were discarded, pellets were resuspended in 190 μL of ice cold wash buffer (20 mM Tris-HCl, pH 7.2, 100 mM KCl, 1 mM DTT), and samples were centrifuged at 3000 *g* for 10 minutes at 4°C; this specific process was performed twice and supernatants were discarded during each step. Final myofibril pellets were resuspended in 200 μL of ice cold storage buffer (20 mM Tris-HCl, pH 7.2, 100 mM KCl, 20% glycerol, 1 mM DTT) and frozen at -80°C. Sarcoplasmic protein content was determined in duplicate using a cuvette-based BCA assay protocol (Thermo Fisher Scientific) whereby a large volume of sample was assayed (100 μL of 5× diluted sample + 900 μL Reagent A + B). The average CV for duplicate readings was 0.58%, and sarcoplasmic protein concentrations were normalized to input dry muscle weights.

#### Electrophoretic determination of myosin and actin content

Determination of myosin and actin content were performed as previously described by our laboratory [19] and others [25]. Briefly, SDS-PAGE preps were made using 10 μL resuspended myofibrils, 65 μL distilled water (diH_2_O), and 25 μL 4x Laemmli buffer. Samples (15 μL) were then loaded on pre-casted gradient (4–15%) SDS-polyacrylamide gels (Bio-Rad Laboratories) and subjected to electrophoresis at 200 V for 40 minutes using pre-made 1x SDS-PAGE running buffer (Ameresco). Following electrophoresis gels were rinsed in diH_2_O for 15 minutes, and immersed in Coomassie stain (LabSafe GEL Blue; G-Biosciences; St. Louis, MO, USA) for 2 hours. Thereafter, gels were destained in diH_2_O for 60 minutes, bright field imaged using a gel documentation system (UVP), and band densities were determined using associated software. Given that a standardized volume from all samples were loaded onto gels, myosin and actin band densities were normalized to input muscle weights for relative expression. Our laboratory has reported that this method yields exceptional sensitivity in detecting 5–25% increases in actin and myosin content [19]. Notably, actin and myosin content were the only two myofibrillar protein targets of interest given that the combination of these proteins make up a majority (~70%) of the myofibrillar protein pool [20].

#### Proteomic analysis of the sarcoplasmic fractions from PRE and W6

Proteomics analysis was performed on the sarcoplasmic fraction for n = 12 fCSA responders in order to interrogate the sarcoplasmic proteins which were altered from PRE to W6. Each protein (90 μg) sample for triplicate technical runs (30 μg for each run) was prepared for LC-MS/MS analysis using EasyPep Mini MS Sample Prep Kit (Thermo Fisher Scientific). In brief, protein sample was transferred into a new microcentrifuge tube and the final volume was adjusted to 100 μL with general cell lysis buffer (Cell Signaling). Reduction solution (50 μL) and alkylation solution (50 μL) were added to the sample, gently mixed, and incubated at 95°C using heat block for 10 minutes to reduce and alkylate samples. After incubation, the sample was removed from the heat block and cooled to room temperature. The reconstituted enzyme Trypsin/Lys-C Protease Mix solution (50 μL) was added to the reduced and alkylated protein sample and incubated with shaking at 37°C for 2 hours to digest the protein sample. Digestion stop solution (50 μL) was added to the sample and peptides were cleaned using peptide clean-up column according to the kit instructions.

An externally calibrated Thermo Q Exactive HF (high-resolution electrospray tandem mass spectrometer) was used in conjunction with Dionex UltiMate3000 RSLC Nano System (Thermo Fisher Scientific). Sample (5 μL) was aspirated into a 50 μL loop and loaded onto the trap column (Thermo μ-Precolumn 5 mm, with nanoViper tubing 30 μm i.d. × 10 cm). The flow rate was set to 300 nL/min for separation on the analytical column (Acclaim pepmap RSLC 75 μM × 15 cm nanoviper; Thermo Fisher Scientific). Mobile phase A was composed of 99.9% H_2_O (EMD Omni Solvent; Millipore, Austin, TX, USA), and 0.1% formic acid and mobile phase B was composed of 99.9% acetonitrile and 0.1% formic acid. A 60 min linear gradient from 3% to 45% B was performed. The LC eluent was directly nanosprayed into the mass-spectrometer. During chromatographic separation, the Q-Exactive HF was operated in a data-dependent mode and under direct control of the Thermo Excalibur 3.1.66 (Thermo Fisher Scientific). The MS data were acquired using the following parameters: 20 data-dependent collisional-induced-dissociation (CID) MS/MS scans per full scan (350 to 1700 m/z) at 60,000 resolution. MS2 were acquired in centroid mode at 15,000 resolution. Ions with a single charge or charges more than 7 as well as unassigned charge were excluded. A 15-second dynamic exclusion window was used. All measurements were performed at room temperature, and three technical replicates were run for each sample. The raw files were analyzed using Proteome Discoverer (version 2.0, Thermo Fisher Scientific) software package with SequestHT and Mascot search nodes using species specific tremble fasta database and the Percolator peptide validator. The resulting .msf files were further analyzed by the proteome validator software Scaffold v4.4 (Portland, OR, USA). Protein identification was provided as official gene symbols sourced from the National Center for Biotechnology Information (NCBI), and differentially expressed proteins from PRE to W6 (unadjusted p-value <0.05) were analyzed for functionally annotated pathways using the OFFICIAL_GENE_SYMBOL query in DAVID v6.8 [26] as well as Kyoto Encyclopedia of Genes and Genomes (KEGG) pathway analysis package [27]. All raw proteomic data from the PRE and W6 time points are provided in S2 File.

### Immunohistochemistry for fCSA and phalloidin staining

Methods for cryostat sectioning have been employed previously in our laboratory and described elsewhere [21, 22, 28, 29]. Briefly, sections from OCT‐preserved samples were cut at a thickness of 8 μm using a cryotome (Leica Biosystems; Buffalo Grove, IL, USA) and were adhered to positively-charged histology slides. Once all samples were sectioned, microscope slides were stored at -80°C until batch processing occurred for immunohistochemistry.

For mean fCSA determination, sections were air-dried at room temperature for 10 minutes, permeabilized in a phosphate‐buffered saline (PBS) solution containing 0.5% Triton X‐100, and blocked with 100% Pierce Super Blocker (Thermo Fisher Scientific) for 10 minutes. Sections were then incubated for 15 minutes with a pre-diluted commercially-available rabbit anti-dystrophin IgG antibody solution (catalog #: GTX15277; Genetex Inc.; Irvine, CA, USA) and spiked in mouse anti-myosin I IgG (catalog #: A4.951 supernatant; Hybridoma Bank, Iowa City, IA, USA; 40 μL added per 1 mL of dystrophin antibody solution). Sections were then washed for 2 minutes in PBS and incubated in the dark for 15 minutes with a secondary antibody solution containing Texas Red-conjugated anti-rabbit IgG (catalog #: TI-1000; Vector Laboratories, Burlingame, CA, USA), and Alexa Fluor 488-conjugated anti-mouse IgG (catalog #: A-11001; Thermo Fisher Scientific; 6.6 μL of all secondary antibodies per 1 mL of blocking solution). Sections were washed for 2 minutes in PBS, air-dried and mounted with fluorescent media containing 4,6-diamidino- 2-phenylindole (DAPI; catalog #: GTX16206; Genetex Inc.). Following mounting, slides were stored in the dark at 4°C until fluorescent images were obtained. After staining was performed on all sections, digital 10× objective images were captured using a fluorescence microscope (Nikon Instruments, Melville, NY, USA). All images were captured by a laboratory technician who was blinded to the labeling codes on microscope slides. Approximate exposure times were 400 ms for TRITC and FITC imaging, and 80 ms for DAPI imaging. This staining method allowed the identification of cell membranes (detected by the Texas Red filter), type I fiber green cell bodies (detected by the FITC filter), type II fiber black cell bodies (unlabeled), and myonuclei (detected by the DAPI filter). Combined CSA measurements of both fiber types (mean fCSA) were performed using custom-written pipelines in the open-sourced software CellProfiler [30] whereby the number of pixels counted within the border of each muscle fiber was converted to a total area (μm^2^). A calibrator slide containing a 250,000 μm^2^ square image was also captured, and pixels per fiber from imaged sections were converted to area using this calibrator image. On average, 113±26 fibers per cross-section were identified for analysis at each sampling time. A post hoc experiment performed in our laboratory to examine potential differences in fCSA measurements between sections on the same slide (n = 26 slides) revealed strong reliability using this method (ICC = 0.929).

For actin protein content determination per fiber cross section, F-actin labelling using Alexa Fluor 488-conjugated (AF488) phalloidin was performed according to previous reports to estimate actin protein content per muscle fiber cross section [31, 32]. Briefly, sections were air-dried for 10 minutes and fixed in formalin for 10 minutes. Sections were then washed with PBS for 5 minutes, and blocked with 100% Pierce Super Blocker (Thermo Fisher Scientific) for 25 minutes. After blocking, a solution containing 5% Super Blocker, 1% rabbit anti-human collagen I IgG (catalog #: ab34710; Abcam, Cambridge, MA, USA), and 3% phalloidin-AF488 (catalog #: A12379; Thermo Fisher Scientific) was placed on sections in the dark for 30 minutes. Sections were subsequently washed for 5 minutes with PBS and incubated in the dark for 20 minutes with a secondary antibody solution containing Texas Red-conjugated anti-rabbit IgG (catalog #: TI-1000; Vector Laboratories; ~6.6 μL secondary antibody per 1 mL of blocking solution). Sections were washed for 5 minutes in PBS, air-dried and mounted with fluorescent media (Genetex Inc.). Following mounting, slides were stored in the dark at 4°C until immunofluorescent images were obtained. After staining was performed on all sections, digital images were captured from blinded samples using a fluorescence microscope (Nikon Instruments) using the 20× objective. Exposure times were 80 milliseconds for FITC and 2 seconds for TRITC imaging. This staining method allowed the identification of actin protein (detected by the FITC filter) and outer cell matrix (detected by the Texas Red filter). Automated measurements of phalloidin-stained pixel intensity per fiber were performed using CellProfiler whereby the number of pixels counted within the border of each muscle fiber were converted summed to provide an integrated intensity value. This value was then divided by fiber area in order to derive a relative contractile intensity value per fiber per μm^2^.

### Serum analysis

Upon blood collection, serum tubes were centrifuged at 3,500 *g* for 5 minutes at room temperature. Aliquots were then placed in 1.7 mL polypropylene tubes and stored at -80°C until batch-processing. An activity assay was used to determine serum levels of CK (Bioo Scientific, Austin, TX, USA). All kits were performed according to manufacturer's instructions and plates were read using a 96-well spectrophotometer (BioTek, Winooski, VT, USA). Due to missed time points and one subject demonstrating exceptionally high PRE serum CK activity values, only 11 subjects were included in the PRE-W6 analysis, and only 6 subjects were included in the W7 analysis described below.

### W7 biopsy assays from a subset of fCSA responders

VL biopsies were obtained from subset of fCSA responders (n = 7) 8 days following the last training bout (termed W7). These individuals refrained from training between W6 and W7, and the purpose of this visit was to ensure that training-induced damage had subsided following transient deloading. Due to financial constraints, only select assays were performed on these subjects and are presented in the results.

### Statistical analysis

Dependent variable comparisons over time were analyzed using one-way repeated measures ANOVAs with LSD post hoc tests. When sphericity was violated (Mauchly's Test of Sphericity p<0.05), Greenhouse-Geisser corrections were applied. Pearson correlations were also performed on select variables. All statistical analyses were performed using SPSS v22.0 (IBM Corp, Armonk, NY, USA) and significance was established at p<0.05, although p-values “approaching significance” (i.e., 0.050<p<0.100) were also discussed given the exploratory nature of the investigation. There was inadequate VL biopsy tissue for certain assays or sections for phalloidin staining. Additionally, only 12 participants were analyzed using proteomics analysis due to financial constraints. Thus, participant number for each dependent variable are presented in figures. All raw data can be found in the supporting information section (S1 File).

## Results

### PRE, W3, and W6 alterations in VL biopsy molecular variables in fCSA responders

**Fig 1:**
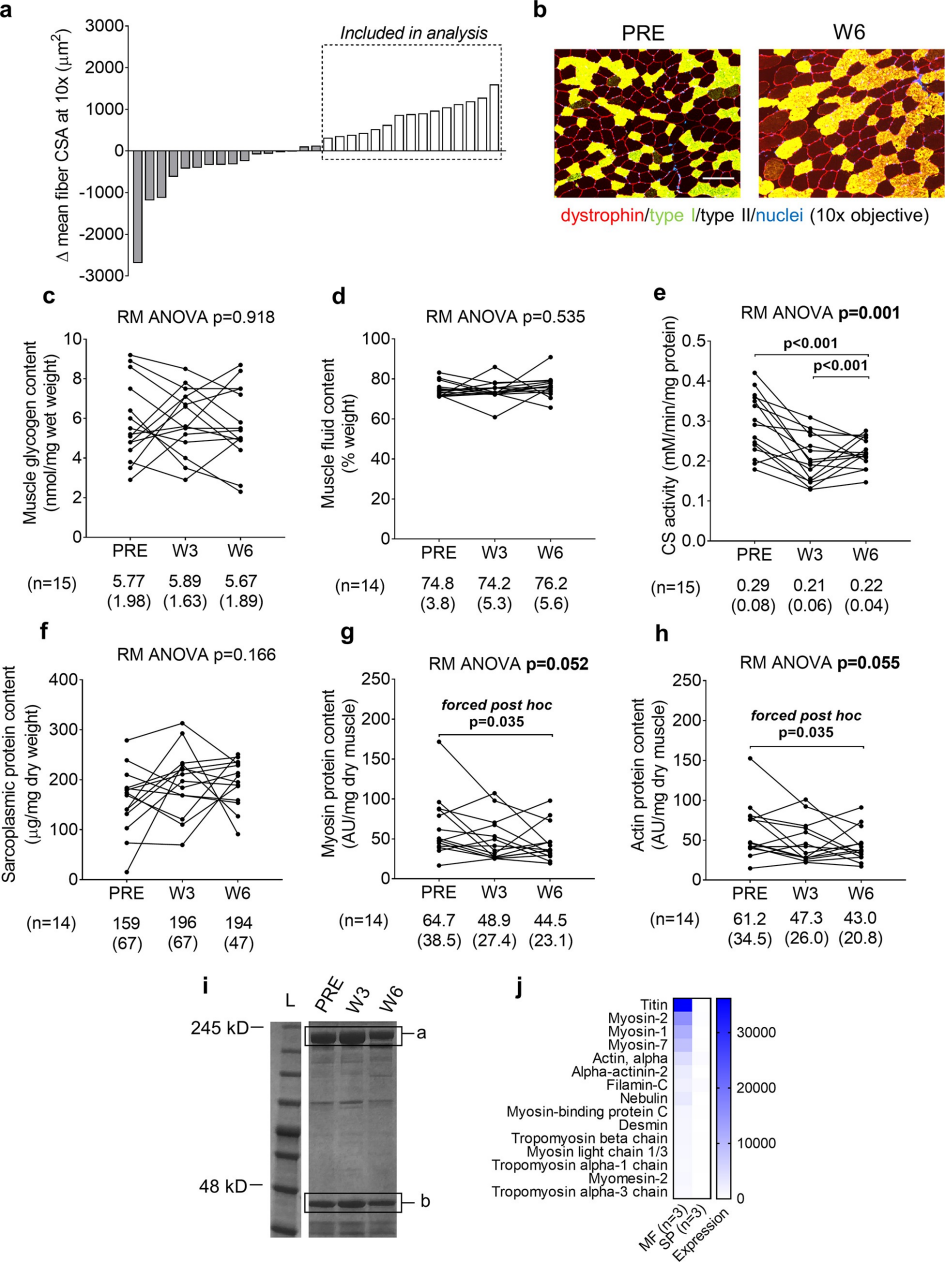
Right leg body composition and VL biopsy molecular adaptations in fCSA responders. All line graph data show individual responses with mean ± standard deviation (SD) values below each time point. Panel a demonstrates the 15 hypertrophic responders according to fCSA changes with training. Panel b illustrates a PRE and W6 representative 10× objective image from one subject (scale bar = 200 μm). No significant changes in VL biopsy glycogen concentrations (panel c), muscle fluid content (panel d), or sarcoplasmic protein concentrations (panel f) were observed. VL biopsy citrate synthase activity (a surrogate of mitochondrial volume) significantly decreased over time (panel e). VL biopsy myosin protein (panel g) and actin protein (panel h) concentrations significantly decreased from PRE to W6. Panel i illustrates representative myosin (band a) and actin (band b) Coomassie stains from one subject. Panel j demonstrates that the utilized myofibrillar and sarcoplasmic isolation methods demonstrated excellent enrichment of contractile proteins in the former fraction, and little contamination of contractile proteins in the latter fraction (both fractions from n = 3 random participants were assayed).

Fig 1A and 1B illustrates the 15 hypertrophic responders according to fCSA changes from PRE to W6. There were no significant changes in VL biopsy glycogen concentrations (Fig 1C), fluid content (Fig 1D), or sarcoplasmic protein concentrations (Fig 1F). Citrate synthase activity decreased from PRE to W3 (p<0.001), and activity also remained lower than PRE at W6 (p<0.001) (Fig 1E). There were no statistically significant changes in myosin and actin protein concentrations (p = 0.052 and p = 0.055, respectively; Fig 1G–1I). However, given that both p-values were approached statistical significance, “forced” post hoc tests were performed. Interestingly, these forced post hoc tests indicated myosin and actin protein concentrations significantly decreased from PRE to W6 (p = 0.035 for each target). Fig 1J demonstrates that the utilized myofibrillar and sarcoplasmic isolation methods show excellent enrichment of contractile proteins in the former fraction, and little contamination of contractile proteins in the latter fraction.

### PRE, W3, and W6 VL biopsy actin content per muscle fiber assessed through phalloidin-AF488 staining in fCSA responders

**Fig 2:**
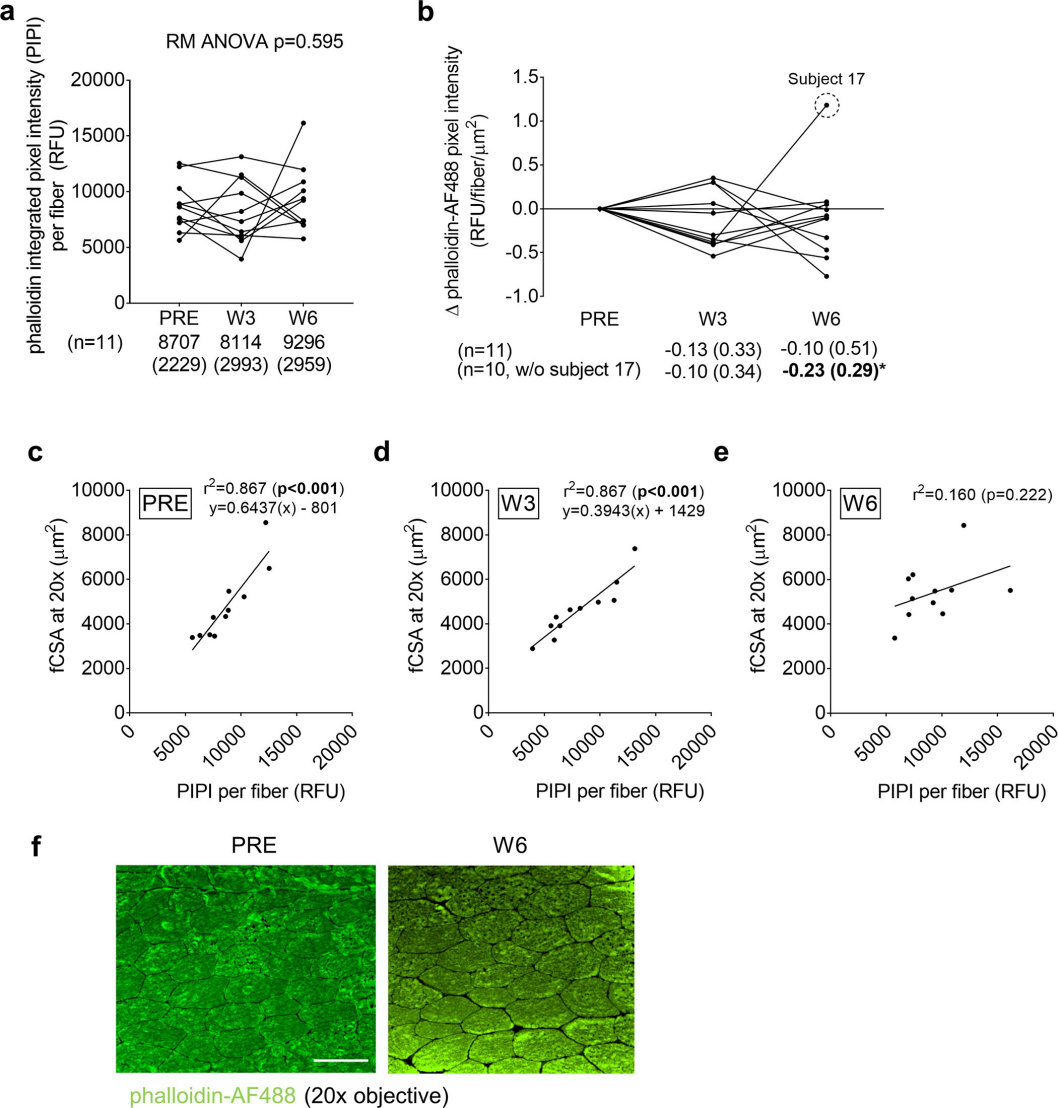
PRE, W3, and W6 VL biopsy contractile protein per muscle fiber assessed through phalloidin-AF488 staining. All line graph data show individual responses with mean ± standard deviation (SD) values below each time point. Phalloidin-AF488 integrated pixel intensity (PIPI) per fiber represents the amount of actin per fiber where increases in this metric suggest increases in the amount of actin per muscle fiber. This metric was not significantly altered throughout the intervention (panel a). Phalloidin pixel intensity per fiber per μm^2^, which represents actin content corrected for fCSA, demonstrated numerical but non-significant decreases from PRE to W3 and W6 (panel b), although the removal of subject 17 (statistical outlier) indicated a significant decrease occurred from PRE to W6. Panels c and d demonstrate a tight coupling at PRE and W3 between fCSA and contractile protein content per fiber. However, only a modest and non-significant correlation was observed at W6 (panel e). Panel g illustrates PRE and W6 phalloidin-AF488 stain 20× images from one subject (scale bar = 100 μm).

Fig 2 demonstrates phalloidin-AF488 staining results of 11 of the 15 subjects who yielded muscle tissue of adequate quality for staining. Phalloidin integrated pixel intensity (PIPI) per fiber represents the amount of actin per fiber cross-section, and an increase in this metric with training indicates muscle fiber actin accretion. Interestingly, PIPI was not significantly altered in these 11 fCSA responders throughout the intervention indicating there was no significant actin accretion (Fig 2A). Phalloidin pixel intensity per fiber per μm^2^ represents contractile protein density (or concentration), and no changes in this metric would indicate that actin accretion occurs in proportion to muscle fiber growth whereas a decrease in this metric would indicate a reduction in actin concentration. The data in Fig 2B represent the W3 and W6 change scores in this metric from the PRE time point. There were numerical but non-significant decreases from PRE to W3 (p = 0.226) and W6 (p = 0.519). However, the removal of subject 17 (statistical outlier >2 SD from mean) indicated a significant decrease occurred from PRE to W6 (p = 0.020). Another insightful analysis included examining the association between fCSA and PIPI, where a very high association in this variable indicates the coupling of fiber size and actin content. There was tight coupling at PRE (r^2^ = 0.815, p<0.001; Fig 2C) and W3 (r^2^ = 0.867, p<0.001; Fig 2D) suggesting a proportional relationship existed between fCSA and actin content per fiber at these time points. However, a weak non-significant correlation was observed at W6 (r^2^ = 0.160, p = 0.222; Fig 2E) suggesting a disproportionate increase in fCSA occurred without increases in actin content per fiber. Fig 2F demonstrates a representative phalloidin-AF488 staining image from a participant at the PRE and W6 time points.

### PRE, W3, and W6 VL biopsy and serum markers of proteolysis and damage in fCSA responders

**Fig 3:**
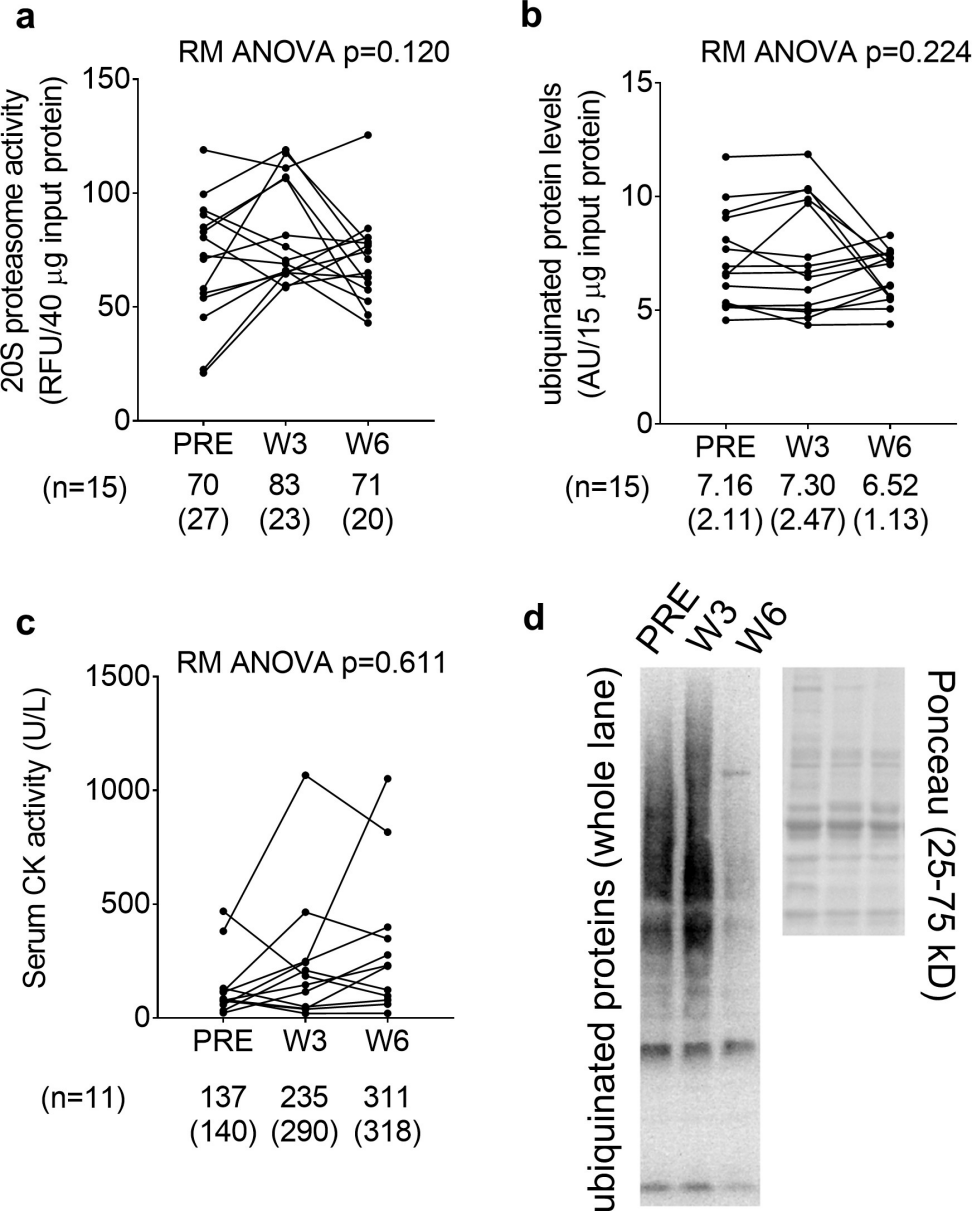
PRE, W3, and W6 VL biopsy proteolysis and muscle damage markers. All line graph data show individual responses with mean ± standard deviation (SD) values below each time point. 20S proteasome activity (panel a), ubiquinated protein levels (panel b), and serum CK activity (panel c) did not significantly change over time. Panel d illustrates a representative Western blot image of ubiquinated protein levels from one subject.

Given that actin and myosin concentrations decreased from PRE to W6, we were interested in examining if markers of proteolysis or damage were concomitantly elevated. However, 20S proteasome activity, ubiquinated protein levels, and serum CK activity did not significantly change over time (Fig 3A–3C). Fig 3D demonstrates a representative Western blot image of ubiquinated protein levels from a participant at the PRE, W3, and W6 time points.

### Proteomics analysis of the PRE and W6 sarcoplasmic fractions from fCSA responders

**Fig 4:**
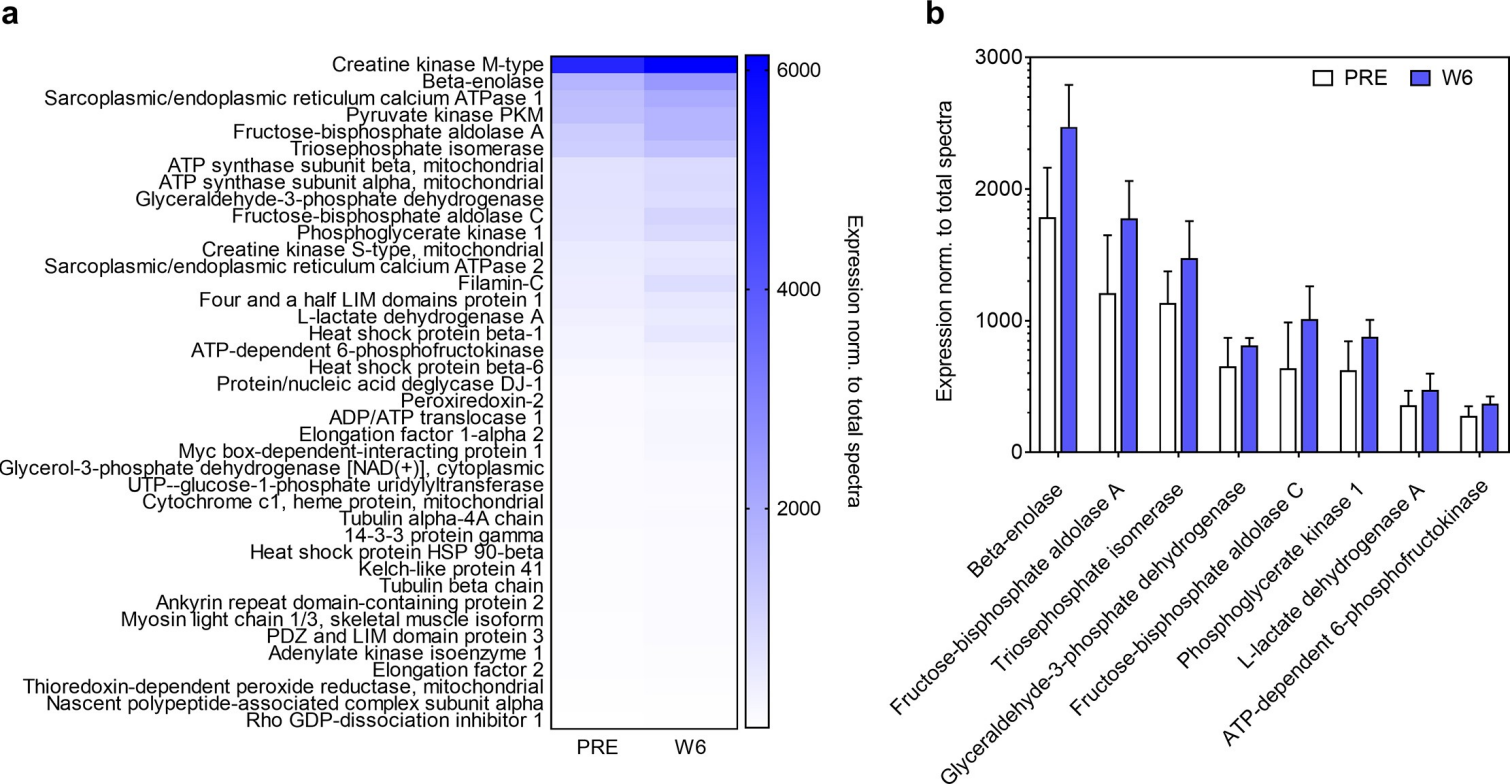
PRE and W6 sarcoplasmic fraction proteomic analysis. Proteomics analysis was performed by LC-MS/MS on n = 12 participants at PRE and W6. Out of the 157 sarcoplasmic proteins detected using proteomics analysis, 40 proteins were significantly upregulated from PRE to W6 (p<0.05; panel a). KEGG pathway analysis indicated that only one pathway termed glycolysis/gluconeogenesis (8 up-regulated enzymes) was significantly up-regulated from PRE to W6 (panel b). Data in panel b are presented as mean ± standard deviation values.

Given that sarcoplasmic protein concentrations numerically increased from PRE to W6 and actin and myosin protein concentrations concomitantly decreased, we sought to examine which sarcoplasmic proteins were altered. Of the 157 sarcoplasmic proteins detected at both PRE and W6, 40 proteins were significantly upregulated (p<0.05; Fig 4A), and 1 protein was significantly down-regulated from PRE to W6 (tropomyosin beta chain, 0.29-fold relative to PRE; *data not shown on Fig 4*). KEGG pathway analysis indicated that only one pathway termed glycolysis/gluconeogenesis (8 up-regulated proteins) was significantly up-regulated from PRE to W6 (FDR value 5.9E-6; Fig 4B). Pathway analysis using DAVID v6.8 indicated that the following functionally-annotated pathways were significantly upregulated: a) glycolysis (8 proteins, FDR value = 1.5E-9), b) acetylation (23 proteins, FDR value = 2.6E-8), c) gluconeogenesis (5 proteins, FDR value = 4.0E-5) and d) cytoplasm (20 proteins, FDR value = 8.9E-4).

### W7 VL biopsy variables and serum CK activity levels in a subset of fCSA responders

**Fig 5:**
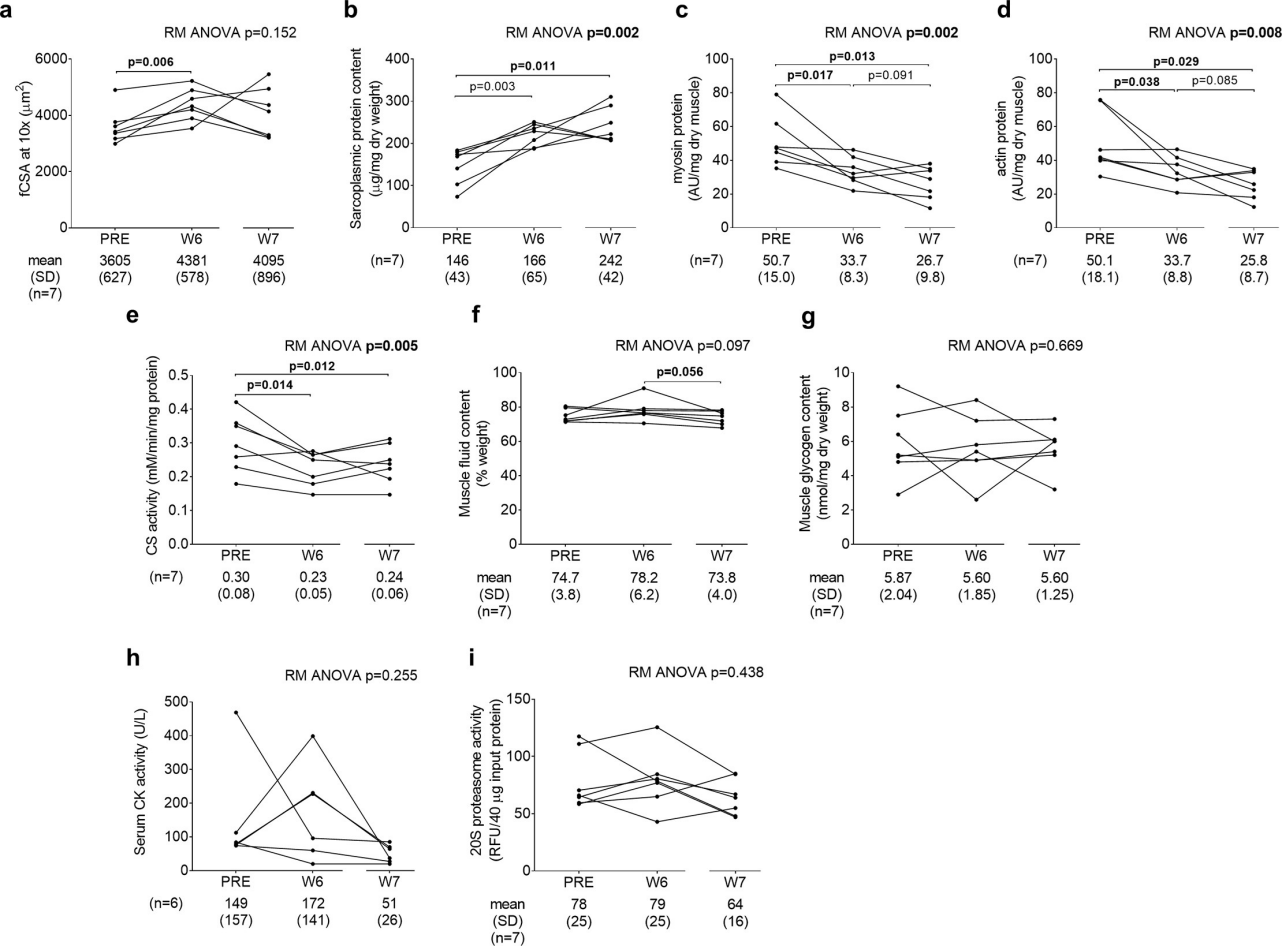
Actin and myosin decrements and concomitant increases in sarcoplasmic protein concentrations persist after one week of detraining. All line graph data show individual responses with mean ± standard deviation (SD) values below each time point. fCSA significantly increased from PRE to W6, although W7 fCSA was not different from PRE or W6 (panel a). Sarcoplasmic protein content significantly increased from PRE to W6, and remained elevated at PRE versus W7 (panel b). Actin and myosin concentrations were significantly lower at W6 and W7 versus PRE, and trended downward from W6 to W7 (panels c and d). CS activity decreased from PRE to W6 and W7 (panel e). While muscle fluid content did not increase from PRE to W6, this metric trended downward from W6 to W7 (panel f). There were no significant changes in muscle glycogen concentrations (panel g), serum CK activity (panel h) or muscle 20S proteasome activity (panel i).

Fig 5 demonstrates data from the 7 fCSA responders that refrained from training 8 days following the last training bout. The repeated measures ANOVA p-value for mean fCSA was 0.152 (Fig 5A). Given that the n-size was limited for these data we performed a forced post hoc and confirmed that fCSA increased from PRE to W6 in these 7 fCSA responders (p = 0.006). However, W7 fCSA was not significantly different from PRE (p = 0.326) or W6 (p = 0.515). Sarcoplasmic protein content significantly increased from PRE to W6 (p = 0.003), and remained elevated at PRE versus W7 (p = 0.011) (Fig 5B). Myosin and actin concentrations were significantly lower at W6 and W7 versus PRE (p<0.05 for both targets), and trended downward from W6 to W7 (p<0.10 for both targets) (Fig 5C and 5D). CS activity decreased from PRE to W6 and W7 (p<0.05 at both levels of time) (Fig 5E). Interestingly, while muscle fluid content did not increase from PRE to W6, this metric trended downward from W6 to W7 (p = 0.056; Fig 5F). There were no significant changes in muscle glycogen concentrations (Fig 5G), serum CK activity (Fig 5H) or muscle 20S proteasome activity (Fig 5I).

### Select biochemical analyses in non-fCSA responders at the PRE, W3, and W6 time points

As shown in Fig 1A (gray bars), n = 15 participants seemed to exhibit no meaningful change (within -82 to 132 μm^2^) or a decrease in mean fCSA (>300 μm^2^) from PRE to W6. While the full complement of assays were not performed in these individuals, data from select biochemical assays in these non-fCSA responders are presented in S1 Fig. Similar to fCSA responders, there were significant changes in myosin and actin protein concentrations (p = 0.002 for each target; panels a and b in S1 Fig), and post hoc tests indicated myosin and actin protein concentrations significantly decreased from PRE to W3 (p = 0.021 and p = 0.020, respectively) and PRE to W6 (p = 0.009 and p = 0.007, respectively). Unlike fCSA responders, there was a significant decrease in ubiquinated protein levels from PRE to W6 (p = 0.034; panel d in S1 Fig). No significant changes were observed for sarcoplasmic protein concentrations (repeated measures ANOVA p = 0.336; panel c in S1 Fig), 20S proteasome activity (repeated measures ANOVA p = 0.306; panel e in S1 Fig), or muscle glycogen concentrations (repeated measures ANOVA p = 0.591; panel F in S1 Fig).

## Discussion

There is a widespread consensus that resistance training-induced skeletal muscle hypertrophy involves mean fCSA increases due to proportional increases in contractile proteins. In this regard, several reports have implied that the addition of sarcomeres in parallel and/or the addition of new myofibrils are largely responsible for muscle fiber hypertrophy [33–35]. This hypothesis seems logical given that elegant tracer studies have consistently demonstrated that high-volume resistance exercise increases MyoPS days following or weeks during the training stimulus. Notwithstanding, and as discussed earlier, several studies suggest skeletal muscle myofibril protein density may actually decrease following weeks to years of higher volume resistance training in humans [16–18]. Furthermore, a recent study suggests that lower specific tensions are evident in single fibers isolated from bodybuilders with significantly larger fCSAs relative to control and power athlete fibers [36]. These authors explicitly stated “*hypertrophy has a detrimental effect on specific tension*”, and noted that myofibril dilution through higher volume body building-style training may have been a driving factor for their observations.

In the current study, an intriguing observation was that actin and myosin concentrations decreased in lieu of fCSA increases over 6 weeks of high-volume resistance training in previously-trained participants. Phalloidin staining verified that actin content per fiber did not change in lieu of hypertrophy, and suggests that actin filament density decreased. Additionally, a dilution effect in mitochondrial content was also observed through observed decrements in muscle CS activity. It is possible that the extraordinarily high training volumes may have accelerated muscle proteolysis or muscle damage leading to a loss of actin, myosin and mitochondrial proteins. While proteolysis rates were not determined at each time point, muscle 20S proteasome activity and poly-ubiquinated protein levels (surrogates of proteolysis) were not significantly different from baseline at the time points subsequent samples were collected. However, this does not discount the possibility proteolytic rates, or other proteolytic systems (e.g., calpain and autophagy) were transiently increased at time points prior to or following inquiry and the underlying mechanisms of our findings warrant further research. Moreover, there was a numerical increase in sarcoplasmic protein concentrations from PRE to W6 that trended towards significance (p = 0.065; data not shown due to RM ANOVA p>0.100), and a significant increase in sarcoplasmic protein concentrations from PRE to W7 in the subset of fCSA responders that donated a biopsy 8 days following the last training bout. Collectively, these data suggest that: a) sarcoplasmic expansion (i.e., sarcoplasmic hypertrophy) was the primary mode through which muscle hypertrophy occurred from PRE to W6, and b) this effect seemed to persist up to 8 days following the last training bout in the subset of participants that were analyzed at the W7 time point.

Our interpretation of data presented in Figs 1 and 2 was that sarcoplasmic expansion occurred from PRE to W6. Thus, we elected to perform a proteomic analytical approach of the sarcoplasmic protein fractions at these time points. Bioinformatics suggested that there was a significant up-regulation of numerous enzymes involved in glycolysis from PRE to W6. Aside from this pathway, it is also notable that several enzymes involved in ATP generation were also upregulated including two creatine kinase isoforms (cytosolic and mitochondrial), two ATP synthase subunits (alpha and beta), ADP/ATP translocase, and adenylate kinase. In an attempt to logically harmonize our findings with existing knowledge pertaining to muscle physiology, our interpretations of these observations are as follows: a) high-volume resistance training may promote sarcoplasmic expansion to spatially prime cells for the expansion of the myofibril pool, b) an increase in the expression of enzymes involved in ATP re-synthesis and metabolism is sensible considering that myofibrillar protein expansion requires large amounts of ATP due to the energetic costs of synthesizing and assimilating myofibrillar proteins, and c) an upregulation in sarcoplasmic proteins related to ATP generation and excitation-contraction coupling may also occur in lieu of sarcoplasmic expansion due to the metabolic demands of high-volume training as well as the energetic demands of myofibrillar protein synthesis and assimilation. Given that these subjects were previously trained, and fCSA values prior to training may have been approaching peak values relative to each individual, it is also possible that hypertrophic growth through sarcoplasmic expansion in response to high volume training may be a result of prior training status. Alternatively stated, fCSA increases with high volume training in untrained individuals may lead to proportional increases in contractile protein, whereas fCSA increases with high volume training in previously trained individuals may lead to disproportional alterations in contractile protein relative to sarcoplasmic expansion. All of the hypothetical scenarios mentioned above hold exciting potential, and studies aimed at addressing these hypotheses are needed.

Considering these findings, we posit that a reappraisal of dogma regarding high-volume resistance training-induced hypertrophy is warranted. In this regard, some have argued that the utilization of high-volume training to volitional fatigue promotes skeletal muscle hypertrophy to a similar degree when compared to lower volume/higher load training [37]. This argument is supported by numerous studies which have determined that both training modalities similarly increase surrogates of skeletal muscle hypertrophy [1, 38–40]. However, these conclusions have primarily been drawn through the utilization of ultrasound to assess muscle thickness or fCSA assessments using histological staining techniques, and none of these studies have examined if the stoichiometric relationship between myofibrillar and sarcoplasmic protein concentrations are preserved relative to pre-training levels. Stated differently, it is unclear in these studies the specific mode through which hypertrophy occurred (e.g., sarcoplasmic, myofibrillar, or connective tissue) and what the functional outcomes of these findings may be. It is also notable that higher load training consistently increases strength compared to lower load training [1, 38–40]. While speculative, it is possible that higher load training (e.g., 3–5 RM lifting) proportionally increases myofibrillar protein levels and fCSA, and this mode of ultrastructural hypertrophy promotes optimal strength gains. Conversely, higher volume/lower load training (e.g., 8–12+ RM lifting) may increase fCSA predominantly through sarcoplasmic expansion and metabolic conditioning, thus leading to sub-optimal strength gains. However, it stands to reason that a combination or specific sequence of the two training emphases (e.g., light-load, high-volume versus heavy-load, low-volume) may produce superior strength and hypertrophy gains compared to the chronic completion of only one or the other (e.g., training periodization). Indeed, as posited by Stone et al. [41] decades ago, it is possible, if not likely, that higher-volume/lower-load training resulting in sarcoplasmic expansion and metabolic conditioning preceding lower-volume/higher-load training can produce more favorable strength outcomes. While these hypotheses are provocative, they are indeed speculative given that the current study lacks a high-load training group and research assessing cellular adaptations with different training styles is lacking. Therefore, studies examining the cellular adaptations to high-load/low-volume versus low-load/high-volume resistance training are warranted.

The implemented training volume was extraordinarily high and the W6 biopsy was collected 24 hours following the last training bout. Therefore, it is plausible that training-induced muscle damage and edema may have influenced many of the dependent variables reported herein. However, much of our data discounts edema being a confounding variable which lead to a decrease in actin and myosin concentrations. First, tissue fluid levels remained unaltered from PRE to W6, and had edema been a confounder then it is plausible that fluid levels would have increased. Additionally, multiple studies discussed above have reported that higher volume resistance training promotes sarcoplasmic expansion and, as noted earlier, our findings are in agreement with these reports. High-volume training-induced sarcoplasmic hypertrophy is also supported by a previous study [5] where the authors examined how 90 days of higher volume resistance training (i.e., up to 12 RM lifts) followed by 90 days of detraining affected mean fCSA. Following 90 days of training, fCSA increased 16% from pre-training values. Three days of detraining (which the authors termed ‘D3’) numerically increased fCSA 3% more from the 90-day point (i.e., the last day of training) and 19% from pre-training. However, fCSA decreased back to pre-training values after only 7 additional days of detraining following D3. Finally, and in relation to the current study, the W7 biopsy data obtained herein provides very compelling evidence supporting the role of sarcoplasmic hypertrophy in facilitating the observed fCSA increases given that myosin and actin concentrations decreased from PRE to W7 with a concomitant increase in sarcoplasmic protein.

### Experimental limitations and future directions

As with all studies that interrogate human skeletal muscle specimens, an experimental limitation to the current study is the extrapolation of findings from relatively small biopsy samples. Additionally, only half of the participants exhibited notable increases in mean fCSA (i.e., high fCSA responders), whereas half of the participants did not exhibit a significant change or exhibited a decrease in mean fCSA values (i.e., low fCSA responders). Interestingly, the 15 high fCSA responders began training with a lower average mean fCSA compared to the 15 low fCSA responders (3,715±684 μm^2^ versus 4,361±927 μm^2^, p = 0.038; *data not shown*). In low fCSA responders myosin and actin protein concentrations decreased from PRE to W3 and, similar to high fCSA responders, levels decreased from PRE to W6. Similar to high fCSA responders, proteolysis markers were not significantly elevated in these individuals. Collectively, these data suggest that: a) participants who began the intervention with smaller fibers realized larger fCSA increases in response to the very high, unaccustomed training volumes, and b) individuals that experienced decrements in fCSA with this style of training may have also experienced a loss in actin and myosin protein. Without tracer data, it is difficult to ascertain how mean fCSA levels (on average) decreased from PRE to W6 in low fCSA responders in the presence of contractile protein concentration decrements. We speculate, however, that these surrogate markers of maladaptation in low fCSA responders were likely due to the implementation of an unaccustomed, high volume training protocol, and this research topic should be more thoroughly investigated. It is notable that the current dataset is that it is a follow-up analysis to a previous study with the original intent of determining if whey protein elicited superior fCSA increases compared to a maltodextrin placebo. Indeed, while there were roughly an equal number fCSA responders in the experimental and placebo groups, this does not rule out the potential involvement of whey protein supplementation in the observed responses herein. Thus, future studies with the primary intent of examining how high volume training affects contractile and sarcoplasmic protein concentrations without nutritional supplementation are needed. Another limitation to the current study is that only college-aged males were examined. Thus, our findings may not represent the physiology that occurs in females or older populations. Additionally, we did not perform W3, W6, or W7 strength testing given that the primary intent of the training paradigm was to elicit skeletal muscle hypertrophy and not strength. However, in retrospect, it would have been interesting to observe if strength changes plateaued or even decreased at these time points relative to PRE, and if these changes were associated with decrements in actin and myosin protein concentrations. Finally, it remains to be determined what other myofibrillar proteins (e.g., nebulin, titin) may be expressed to differing degrees in response to various training interventions beyond myosin and actin, which are considered to occupy the majority of the myofibril, and what specific training stimuli instigate the expression of specific myofibrillar proteins.

## Conclusions

These data challenge current dogma suggesting fCSA increases during high-volume resistance training are primarily driven through increases contractile protein content. Instead, we interpret these data to suggest sarcoplasmic hypertrophy is largely responsible for short-term fCSA increases, and this effect can persist up to 8 days following the cessation of training. Our proteomic data suggest that sarcoplasmic proteins upregulated during higher volume training are involved with glycolysis as well as other processes that generate ATP. Future research is needed to determine the significance of sarcoplasmic hypertrophy during high-volume resistance training, or whether different training paradigms affect this process.

## Supporting information

S1 File Complete raw dataset. (XLSX)

S2 File Complete PRE and W6 sarcoplasmic proteomics dataset from fCSA responders. (XLSX)

S1 Fig Pre, W3, and W6 data from participants not included in the fCSA responder analysis. (TIF)

## References

[1] Mitchell CJ, Churchward-Venne TA, West DW, Burd NA, Breen L, Baker SK, et al Resistance exercise load does not determine training-mediated hypertrophic gains in young men. J Appl Physiol (1985). 2012;113(1):71–7. Epub 2012/04/21. 10.1152/japplphysiol.00307.2012 22518835 PMC3404827

[2] Morton RW, Oikawa SY, Wavell CG, Mazara N, McGlory C, Quadrilatero J, et al Neither load nor systemic hormones determine resistance training-mediated hypertrophy or strength gains in resistance-trained young men. J Appl Physiol (1985). 2016;121(1):129–38. Epub 2016/05/14. 10.1152/japplphysiol.00154.2016 27174923 PMC4967245

[3] Verdijk LB, Gleeson BG, Jonkers RA, Meijer K, Savelberg HH, Dendale P, et al Skeletal muscle hypertrophy following resistance training is accompanied by a fiber type-specific increase in satellite cell content in elderly men. J Gerontol A Biol Sci Med Sci. 2009;64(3):332–9. Epub 2009/02/07. 10.1093/gerona/gln050 19196907 PMC2655000

[4] Mobley CB, Haun CT, Roberson PA, Mumford PW, Kephart WC, Romero MA, et al Biomarkers associated with low, moderate, and high vastus lateralis muscle hypertrophy following 12 weeks of resistance training. PLoS One. 2018;13(4):e0195203 Epub 2018/04/06. 10.1371/journal.pone.0195203 29621305 PMC5886420

[5] Kadi F, Schjerling P, Andersen LL, Charifi N, Madsen JL, Christensen LR, et al The effects of heavy resistance training and detraining on satellite cells in human skeletal muscles. J Physiol. 2004;558(Pt 3):1005–12. Epub 2004/06/26. 10.1113/jphysiol.2004.065904 15218062 PMC1665027

[6] Petrella JK, Kim JS, Mayhew DL, Cross JM, Bamman MM. Potent myofiber hypertrophy during resistance training in humans is associated with satellite cell-mediated myonuclear addition: a cluster analysis. J Appl Physiol (1985). 2008;104(6):1736–42. Epub 2008/04/26. 10.1152/japplphysiol.01215.2007 . 18436694

[7] Reidy PT, Fry CS, Igbinigie S, Deer RR, Jennings K, Cope MB, et al Protein Supplementation Does Not Affect Myogenic Adaptations to Resistance Training. Med Sci Sports Exerc. 2017;49(6):1197–208. Epub 2017/03/28. 10.1249/MSS.0000000000001224 28346813 PMC5433887

[8] Campos GE, Luecke TJ, Wendeln HK, Toma K, Hagerman FC, Murray TF, et al Muscular adaptations in response to three different resistance-training regimens: specificity of repetition maximum training zones. Eur J Appl Physiol. 2002;88(1–2):50–60. Epub 2002/11/19. 10.1007/s00421-002-0681-6 . 12436270

[9] Schuenke MD, Herman JR, Gliders RM, Hagerman FC, Hikida RS, Rana SR, et al Early-phase muscular adaptations in response to slow-speed versus traditional resistance-training regimens. Eur J Appl Physiol. 2012;112(10):3585–95. Epub 2012/02/14. 10.1007/s00421-012-2339-3 . 22328004

[10] Staron RS, Leonardi MJ, Karapondo DL, Malicky ES, Falkel JE, Hagerman FC, et al Strength and skeletal muscle adaptations in heavy-resistance-trained women after detraining and retraining. J Appl Physiol (1985). 1991;70(2):631–40. Epub 1991/02/01. 10.1152/jappl.1991.70.2.631 . 1827108

[11] Reidy PT, Rasmussen BB. Role of Ingested Amino Acids and Protein in the Promotion of Resistance Exercise-Induced Muscle Protein Anabolism. J Nutr. 2016;146(2):155–83. Epub 2016/01/15. 10.3945/jn.114.203208 26764320 PMC4725426

[12] Damas F, Phillips S, Vechin FC, Ugrinowitsch C. A review of resistance training-induced changes in skeletal muscle protein synthesis and their contribution to hypertrophy. Sports Med. 2015;45(6):801–7. Epub 2015/03/06. 10.1007/s40279-015-0320-0 . 25739559

[13] Kumar V, Atherton P, Smith K, Rennie MJ. Human muscle protein synthesis and breakdown during and after exercise. J Appl Physiol (1985). 2009;106(6):2026–39. Epub 2009/01/24. 10.1152/japplphysiol.91481.2008 . 19164770

[14] Wilkinson DJ, Franchi MV, Brook MS, Narici MV, Williams JP, Mitchell WK, et al A validation of the application of D(2)O stable isotope tracer techniques for monitoring day-to-day changes in muscle protein subfraction synthesis in humans. Am J Physiol Endocrinol Metab. 2014;306(5):E571–9. Epub 2014/01/02. 10.1152/ajpendo.00650.2013 24381002 PMC3948971

[15] Brook MS, Wilkinson DJ, Mitchell WK, Lund JN, Szewczyk NJ, Greenhaff PL, et al Skeletal muscle hypertrophy adaptations predominate in the early stages of resistance exercise training, matching deuterium oxide-derived measures of muscle protein synthesis and mechanistic target of rapamycin complex 1 signaling. FASEB J. 2015;29(11):4485–96. Epub 2015/07/15. 10.1096/fj.15-273755 . 26169934

[16] Penman KA. Ultrastructural changes in human striated muscle using three methods of training. Res Q. 1969;40(4):764–72. Epub 1969/12/01. . 5262107

[17] MacDougall JD, Sale DG, Elder GC, Sutton JR. Muscle ultrastructural characteristics of elite powerlifters and bodybuilders. Eur J Appl Physiol Occup Physiol. 1982;48(1):117–26. Epub 1982/01/01. . 7199447 10.1007/BF00421171

[18] Toth MJ, Miller MS, VanBuren P, Bedrin NG, LeWinter MM, Ades PA, et al Resistance training alters skeletal muscle structure and function in human heart failure: effects at the tissue, cellular and molecular levels. J Physiol. 2012;590(5):1243–59. Epub 2011/12/27. 10.1113/jphysiol.2011.219659 22199163 PMC3381828

[19] Roberts MD, Romero MA, Mobley CB, Mumford PW, Roberson PA, Haun CT, et al Skeletal muscle mitochondrial volume and myozenin-1 protein differences exist between high versus low anabolic responders to resistance training. PeerJ. 2018;6:e5338 Epub 2018/08/02. 10.7717/peerj.5338 30065891 PMC6065464

[20] Haun CT, Vann CG, Roberts BM, Vigotsky AD, Schoenfeld BJ, Roberts MD. A critical evaluation of the biological construct skeletal muscle hypertrophy: size matters but so does the measurement. Front Physiol. 2019 Epub 12 March 2019. 10.3389/fphys.2019.00247. PMC6423469 30930796

[21] Haun CT, Vann CG, Mobley CB, Roberson PA, Osburn SC, Holmes HM, et al Effects of Graded Whey Supplementation During Extreme-Volume Resistance Training. Front Nutr. 2018;5:84 Epub 2018/09/27. 10.3389/fnut.2018.00084 30255024 PMC6141782

[22] Mobley CB, Haun CT, Roberson PA, Mumford PW, Romero MA, Kephart WC, et al Effects of Whey, Soy or Leucine Supplementation with 12 Weeks of Resistance Training on Strength, Body Composition, and Skeletal Muscle and Adipose Tissue Histological Attributes in College-Aged Males. Nutrients. 2017;9(9). 10.3390/nu9090972 28869573 PMC5622732

[23] Larsen S, Nielsen J, Hansen CN, Nielsen LB, Wibrand F, Stride N, et al Biomarkers of mitochondrial content in skeletal muscle of healthy young human subjects. J Physiol. 2012;590(14):3349–60. 10.1113/jphysiol.2012.230185 22586215 PMC3459047

[24] Mobley CB, Mumford PW, Kephart WC, Haun CT, Holland AM, Beck DT, et al Aging in Rats Differentially Affects Markers of Transcriptional and Translational Capacity in Soleus and Plantaris Muscle. Front Physiol. 2017;8:518 10.3389/fphys.2017.00518 28775694 PMC5517446

[25] Cohen S, Brault JJ, Gygi SP, Glass DJ, Valenzuela DM, Gartner C, et al During muscle atrophy, thick, but not thin, filament components are degraded by MuRF1-dependent ubiquitylation. J Cell Biol. 2009;185(6):1083–95. 10.1083/jcb.200901052 19506036 PMC2711608

[26] Huang DW, Sherman BT, Tan Q, Collins JR, Alvord WG, Roayaei J, et al The DAVID Gene Functional Classification Tool: a novel biological module-centric algorithm to functionally analyze large gene lists. Genome Biol. 2007;8(9):R183 Epub 2007/09/06. 10.1186/gb-2007-8-9-r183 17784955 PMC2375021

[27] Kanehisa M, Goto S. KEGG: kyoto encyclopedia of genes and genomes. Nucleic Acids Res. 2000;28(1):27–30. Epub 1999/12/11. 10.1093/nar/28.1.27 10592173 PMC102409

[28] Martin JS, Kephart WC, Haun CT, McCloskey AE, Shake JJ, Mobley CB, et al Impact of external pneumatic compression target inflation pressure on transcriptome-wide RNA expression in skeletal muscle. Physiol Rep. 2016;4(22). 10.14814/phy2.13029 27884954 PMC5357997

[29] Hyatt HW, Toedebusch RG, Ruegsegger G, Mobley CB, Fox CD, McGinnis GR, et al Comparative adaptations in oxidative and glycolytic muscle fibers in a low voluntary wheel running rat model performing three levels of physical activity. Physiol Rep. 2015;3(11). 10.14814/phy2.12619 26603455 PMC4673647

[30] Carpenter AE, Jones TR, Lamprecht MR, Clarke C, Kang IH, Friman O, et al CellProfiler: image analysis software for identifying and quantifying cell phenotypes. Genome Biol. 2006;7(10):R100 10.1186/gb-2006-7-10-r100 17076895 PMC1794559

[31] Duddy W, Duguez S, Johnston H, Cohen TV, Phadke A, Gordish-Dressman H, et al Muscular dystrophy in the mdx mouse is a severe myopathy compounded by hypotrophy, hypertrophy and hyperplasia. Skelet Muscle. 2015;5:16 Epub 2015/05/20. 10.1186/s13395-015-0041-y 25987977 PMC4434871

[32] Gokhin DS, Ward SR, Bremner SN, Lieber RL. Quantitative analysis of neonatal skeletal muscle functional improvement in the mouse. J Exp Biol. 2008;211(Pt 6):837–43. Epub 2008/03/04. 10.1242/jeb.014340 . 18310108

[33] Schoenfeld BJ. The mechanisms of muscle hypertrophy and their application to resistance training. J Strength Cond Res. 2010;24(10):2857–72. Epub 2010/09/18. 10.1519/JSC.0b013e3181e840f3 . 20847704

[34] Brentano MA, Martins Kruel LF. A review on strength exercise-induced muscle damage: applications, adaptation mechanisms and limitations. J Sports Med Phys Fitness. 2011;51(1):1–10. Epub 2011/02/08. . 21297557

[35] Qaisar R, Bhaskaran S, Van Remmen H. Muscle fiber type diversification during exercise and regeneration. Free Radic Biol Med. 2016;98:56–67. Epub 2016/04/02. 10.1016/j.freeradbiomed.2016.03.025 . 27032709

[36] Meijer JP, Jaspers RT, Rittweger J, Seynnes OR, Kamandulis S, Brazaitis M, et al Single muscle fibre contractile properties differ between body-builders, power athletes and control subjects. Exp Physiol. 2015;100(11):1331–41. Epub 2015/09/22. 10.1113/EP085267 . 26388513

[37] Burd NA, Moore DR, Mitchell CJ, Phillips SM. Big claims for big weights but with little evidence. Eur J Appl Physiol. 2013;113(1):267–8. Epub 2012/10/23. 10.1007/s00421-012-2527-1 . 23086296

[38] Jenkins NDM, Miramonti AA, Hill EC, Smith CM, Cochrane-Snyman KC, Housh TJ, et al Greater Neural Adaptations following High- vs. Low-Load Resistance Training. Front Physiol. 2017;8:331 Epub 2017/06/15. 10.3389/fphys.2017.00331 28611677 PMC5447067

[39] Jenkins ND, Housh TJ, Buckner SL, Bergstrom HC, Cochrane KC, Hill EC, et al Neuromuscular Adaptations After 2 and 4 Weeks of 80% Versus 30% 1 Repetition Maximum Resistance Training to Failure. J Strength Cond Res. 2016;30(8):2174–85. Epub 2016/02/06. 10.1519/JSC.0000000000001308 . 26848545

[40] Ogasawara R, Loenneke JP, Thiebaud RS, Abe T. Low-load bench press training to fatigue results in muscle hypertrophy similar to high-load bench press training. Int J Clin Med 2013;(4):114–21.

[41] Stone MH, Wilson D, R. R, Newton H. Physiological basis. Strength Cond J. 1983;5(6):40–64.

