# Supplementary figures and images for: Muscle fiber hypertrophy in response to 6 weeks of high-volume resistance training in trained young men is largely attributed to sarcoplasmic hypertrophy
Source: PLoS One. 2019 Jun 5;14(6):e0215267. doi: 10.1371/journal.pone.0215267 (PMC6550381; doi:10.1371/journal.pone.0215267)

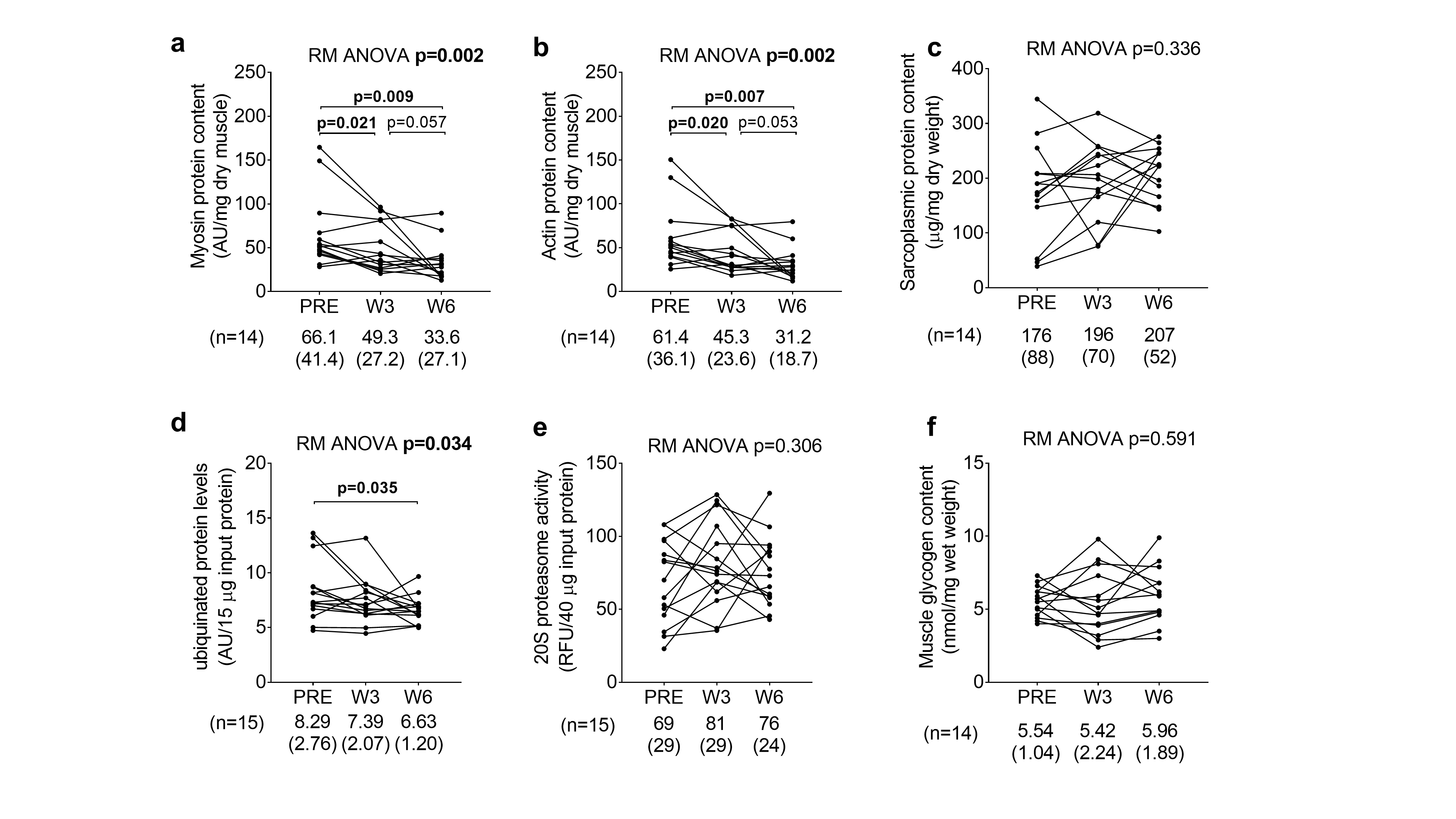

Supplement: S1 Fig — (TIF) [file pone.0215267.s003.tif]
